# 1297. Design and Testing of an Interactive Module for Improving Understanding and Application of Institutional Antibiograms

**DOI:** 10.1093/ofid/ofac492.1128

**Published:** 2022-12-15

**Authors:** Conan MacDougall

**Affiliations:** University of California San Francisco, San Francisco, CA

## Abstract

**Background:**

Many infectious diseases treatment guidelines recommend incorporating local antibiograms (ABs) into treatment decisionmaking. However, studies suggest many healthcare provider trainees are uncomfortable using ABs and rarely incorporate them into their decisionmaking. Our purpose was to develop a brief online module to develop skills in locating and utilizing an institutional AB and to gather feedback on its utility.

Interactive Module for Understanding and Interpreting Antibiograms

**Figure:**
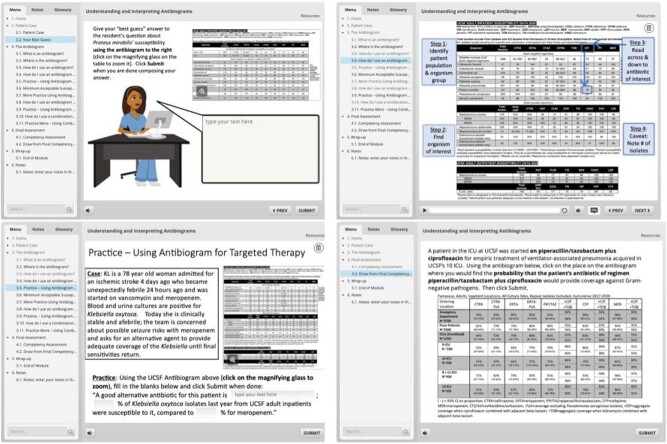

Screen captures displaying initial framing case with learner response (upper left), stepwise navigation of an antibiogram (upper right), embedded task practice (lower left) , and question from final competency assessment (lower right).

**Methods:**

We developed and tested the module at the University of California San Francisco, with initial testing among 340 pharmacy students during microbiology instruction. We used the Four-Component Instructional Design framework to inform our design approach. Using the Articulate Storyline platform, we developed an interactive online learning module (Figure 1). The module began with a patient case to introduce the whole task and activate prior knowledge, with learners required to enter a freetext answer to the case prompt. Using visuals and narration with captions, the module then reviewed the basics of an AB and how to use an AB for therapy with known pathogens and for empiric therapy, and use of location-specific and combination ABs. Task practice was provided by case-based questions requiring learner response and providing immediate feedback. The module ended with competency assessment questions including revisiting the original case and the learner's initial answer. Learners completing the module were directed to complete a survey to provide feedback on the module; we report quantitative and qualitative feedback from the survey.

**Results:**

We received 131 survey responses (38.5%). 111/131 (85%) rated the educational value of the module as “Extremely valuable”, and 103/131 (79%) rated the module as having “Excellent usability”. Primary themes among positive feedback included the interactive elements, stepwise approach, and questions with feedback; constructive feedback included desire for speed control of the narration and sound quality.

**Conclusion:**

An interactive online module for learning about ABs was highly rated by learners and may be valuable to help clinicians apply ABs in practice. Future studies should include other health professions learners, delayed assessment to measure retention, and customization for different institutional ABs.

**Disclosures:**

**Conan MacDougall, PharmD, MAS**, Merck: Advisor/Consultant.

